# (η^6^-Benzene)­(benzyl­diphenyl­phos­phane)dichloridoruthenium(II)

**DOI:** 10.1107/S1600536812037154

**Published:** 2012-09-01

**Authors:** Alfred Muller, Wade L. Davis

**Affiliations:** a Research Centre for Synthesis and Catalysis, Department of Chemistry, University of Johannesburg (APK Campus), PO Box 524, Auckland Park, Johannesburg, 2006, South Africa

## Abstract

In the title compound, [RuCl_2_(C_6_H_6_)(C_19_H_17_P)], the Ru^II^ atom has a distorted pseudo-octa­hedral coordination environment with the metrical parameters around the metallic core as Ru—centroid(η^6^-benzene) = 1.6894 (11) Å, Ru—P = 2.3466 (6), Ru—Cl(avg.) = 2.4127 (7) Å; Cl—Ru—Cl = 88.07 (2) and Cl—Ru—P = 82.77 (2), 87.65 (2)°. The effective cone angle for the benzyl­diphenyl­phosphane was calculated to be 143°. In the crystal C—H⋯Cl and C—H⋯π inter­actions are observed.

## Related literature

For catalytic activity studies on Ru^II^–arene complexes, see: Chen *et al.* (2002 ▶); De Clercq & Verpoort (2002 ▶); Wang *et al.* (2011 ▶); Aydemir *et al.* (2011 ▶). For background to ring-opening metathesis polymerization with Ru–arene complexes, see: Stumpf *et al.* (1995 ▶). For background to cone angles, see: Otto (2001 ▶); Tolman (1977 ▶). For a description of the Cambridge Structural Database, see: Allen (2002 ▶).
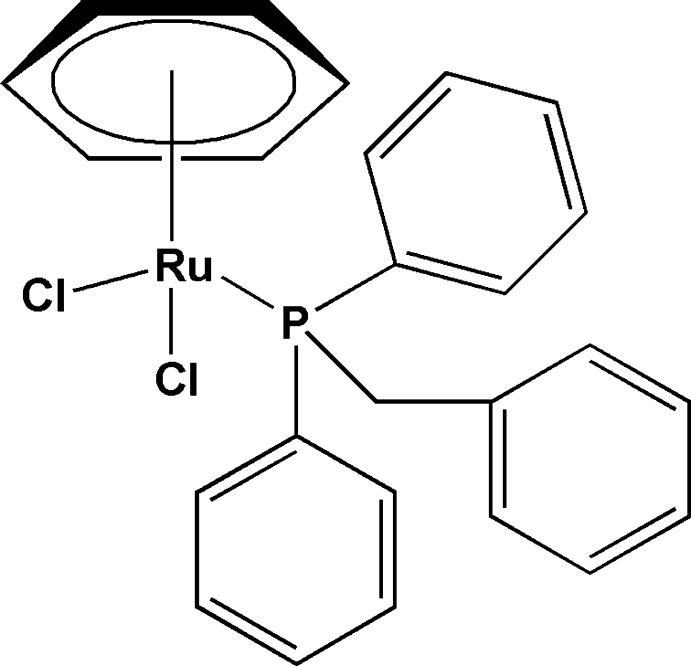


## Experimental

### 

#### Crystal data

[RuCl_2_(C_6_H_6_)(C_19_H_17_P)]
*M* *_r_* = 526.37
Orthorhombic, 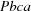

*a* = 16.8415 (9) Å
*b* = 14.1497 (7) Å
*c* = 18.6919 (8) Å
*V* = 4454.3 (4) Å^3^
*Z* = 8
Mo *K*α radiation
μ = 1.03 mm^−1^
*T* = 100 K
0.09 × 0.03 × 0.01 mm

#### Data collection

Bruker APEX DUO 4K-CCD diffractometer
Absorption correction: multi-scan (*SADABS*; Bruker, 2008 ▶) *T*_min_ = 0.429, *T*_max_ = 0.629
34735 measured reflections
5544 independent reflections
4056 reflections with *I* > 2σ(*I*)
*R*_int_ = 0.063

#### Refinement

*R*[*F* ^2^ > 2σ(*F* ^2^)] = 0.032
*wR*(*F* ^2^) = 0.069
*S* = 1.01
5544 reflections
262 parameters
H-atom parameters constrained
Δρ_max_ = 0.44 e Å^−3^
Δρ_min_ = −0.58 e Å^−3^

### 

Data collection: *APEX2* (Bruker, 2011 ▶); cell refinement: *SAINT* (Bruker, 2008 ▶); data reduction: *SAINT* and *XPREP* (Bruker, 2008 ▶); program(s) used to solve structure: *SIR97* (Altomare *et al.*, 1999 ▶); program(s) used to refine structure: *SHELXL97* (Sheldrick, 2008 ▶); molecular graphics: *DIAMOND* (Brandenburg & Putz, 2005 ▶); software used to prepare material for publication: *publCIF* (Westrip, 2010 ▶) and *WinGX* (Farrugia, 1999 ▶).

## Supplementary Material

Crystal structure: contains datablock(s) global, I. DOI: 10.1107/S1600536812037154/bt6823sup1.cif

Structure factors: contains datablock(s) I. DOI: 10.1107/S1600536812037154/bt6823Isup2.hkl

Additional supplementary materials:  crystallographic information; 3D view; checkCIF report

## Figures and Tables

**Table 1:** Hydrogen-bond geometry (Å, °) *Cg*1 and *Cg*2 are the centroids of the C8–C13 and C20–C25 rings, respectively.

*D*—H⋯*A*	*D*—H	H⋯*A*	*D*⋯*A*	*D*—H⋯*A*
C11—H11⋯Cl2^i^	0.95	2.85	3.568 (3)	133
C16—H16⋯Cl1^ii^	0.95	2.8	3.716 (3)	163
C2—H2⋯Cl2^iii^	0.95	2.87	3.733 (3)	151
C24—H24⋯Cl2^iv^	0.95	2.78	3.685 (3)	161
C21—H21⋯*Cg*1^v^	0.95	2.89	3.673 (3)	140
C4—H4⋯*Cg*2^vi^	0.95	3.00	3.789 (3)	141

Symmetry codes: (i) 
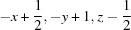
; (ii) 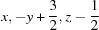; (iii) 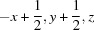; (iv) 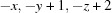; (v) 
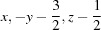
; (vi) 
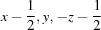
.

**Fig. 1.**
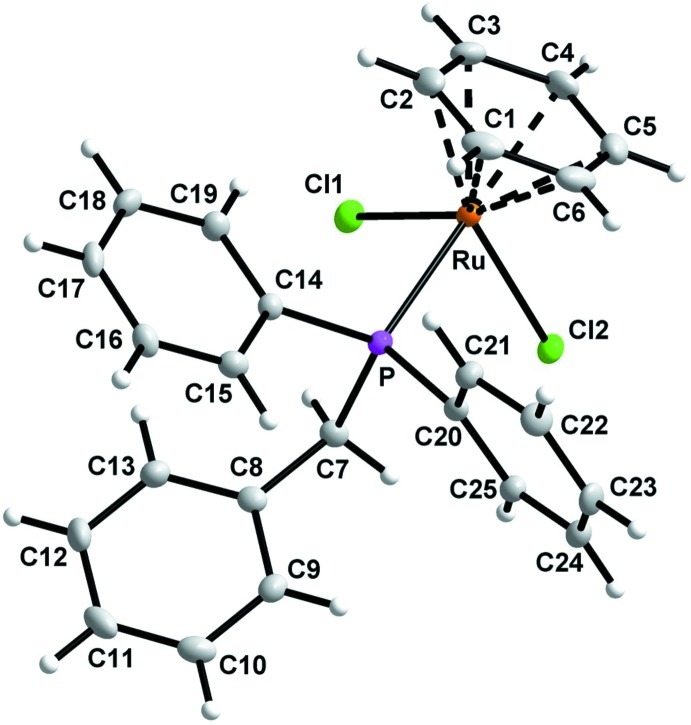
A view of the title complex, (I) showing the atom-numbering scheme and 50% probability displacement ellipsoids.

**Fig. 2.**
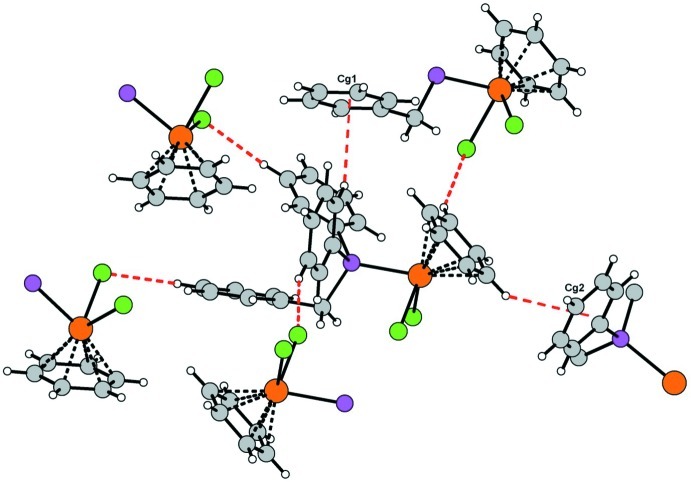
Packing diagram of (I) showing the C–H···Cl and C–H···π interactions (indicated by red dashed lines).

## supplementary crystallographic information

### Comment

The activity of the half-sandwich Ru(II)-arene complexes are well known in the catalytic transfer hydrogenation of carbonyl compounds (Chen *et al.*, 2002; De Clercq & Verpoort, 2002; Wang *et al.*, 2011; Aydemir *et al.*, 2011) and for ring-opening metathesis polymerization (Stumpf *et al.*, 1995). Reported here is the η^6^-Ru compound containing benzyldiphenylphosphane as part of our ongoing investigation into these type of complexes.

Molecules of the title compound packs in the orthorhombic space group Pbca (*Z* = 8), and reveals the typical piano-stool geometry for these complexes. The coordination sphere of the ruthenium is occupied by a benzene, benzyldiphenylphosphane and two chloride atoms (see Fig. 1). The distance between Ru and the centroid of the π-bonded η^6^-benzene ligand is 1.6894 (11) Å and the mean Ru—C bond distance is 2.198 (3) Å. The coordination of the remaining ligands to the Ru atom shows deviation from the typical octahedral geometry with Cl—Ru—Cl = 88.02 (2) and Cl—Ru—P = 82.77 (2), 87.65 (2)°. The bond distances of Ru—P = 2.346 (6) and Ru—Cl(avg.) = 2.412 (6) Å are within normal ranges (Allen, 2002).

To describe the steric demand of phosphane ligands the Tolman cone angle (Tolman, 1977) is still the most commonly used model. Applying this model to the geometry obtained from the title compound (and adjusting the Ru—P bond distance to 2.28 Å) we calculated an effective cone angle (Otto, 2001) of 143°. The small value for the cone angle can be ascribed to the orientation of the benzylic group of the phosphane ligand, pointing away from the metal core. This orientation is fairly rare as a CSD search shows 5 out of 28 hits with this conformation (Allen, 2002; search conducted on all transition metals with this phosphane ligand). The preferred orientation of the benzyldiphenylphosphane ligand could be linked to the number of C–H···Cl and C–H···π interactions associated with it (see Figure 2, Table 1).

### Experimental

[(C_6_H_6_)RuCl_2_]_2_ (50.0 mg, 0.10 mmol) and benzyldiphenylphosphane (60.4 mg, 0.22 mmol) in benzene (25 ml) were refluxed under argon for 4 h. The resulting red solution was filtered, the filtrate concentrated under reduced pressure to *ca* 5 ml whereby a sample suitable for single-crystal X-ray diffraction was obtained as red plates. Analytical data: ^31^P {H} NMR (CDCl_3_, 161.99 MHz): δ (p.p.m.) 30.34 (*s*). ^1^H NMR (CDCl_3_, 400 MHz): δ (p.p.m.) 2.15 (s, 2H, CH_2_); 5.30 (s, 6H, C_6_H_6_); 6.43 (d, 2H, Ar—H of C_7_H_7_); 6.85 (t, 2H, Ar—H of C_7_H_7_); 6.97 (t, 1H, Ar—H of C_7_H_7_); 7.35 (d, 4H, Ar—H of C_6_H_5_); 7.44 (t, 2H, Ar—H of C_6_H_5_); 7.67 (t, 4H, Ar—H of C_6_H_5_).

### Refinement

The aromatic and methylene H atoms were placed in geometrically idealized positions (C—H = 0.95–0.99) and allowed to ride on their parent atoms, with *U*_iso_(H) = 1.2*U*_eq_(C).

### Crystal data

**Table:**

[RuCl_2_(C_6_H_6_)(C_19_H_17_P)]	*F*(000) = 2128
*M**_r_* = 526.37	*D*_x_ = 1.57 Mg m^−^^3^
Orthorhombic, *P**b**c**a*	Mo *K*α radiation, λ = 0.71073 Å
Hall symbol: -P 2ac 2ab	Cell parameters from 5707 reflections
*a* = 16.8415 (9) Å	θ = 2.2–28.1°
*b* = 14.1497 (7) Å	µ = 1.03 mm^−^^1^
*c* = 18.6919 (8) Å	*T* = 100 K
*V* = 4454.3 (4) Å^3^	Plate, red
*Z* = 8	0.09 × 0.03 × 0.01 mm

### Data collection

**Table:**

Bruker APEX DUO 4K-CCD diffractometer	5544 independent reflections
Radiation source: sealed tube	4056 reflections with *I* > 2σ(*I*)
Graphite monochromator	*R*_int_ = 0.063
Detector resolution: 8.4 pixels mm^-1^	θ_max_ = 28.3°, θ_min_ = 2.2°
φ and ω scans	*h* = −10→22
Absorption correction: multi-scan (*SADABS*; Bruker, 2008)	*k* = −18→18
*T*_min_ = 0.429, *T*_max_ = 0.629	*l* = −24→24
34735 measured reflections	

### Refinement

**Table:**

Refinement on *F*^2^	Primary atom site location: structure-invariant direct methods
Least-squares matrix: full	Secondary atom site location: difference Fourier map
*R*[*F*^2^ > 2σ(*F*^2^)] = 0.032	Hydrogen site location: inferred from neighbouring sites
*wR*(*F*^2^) = 0.069	H-atom parameters constrained
*S* = 1.01	*w* = 1/[σ^2^(*F*_o_^2^) + (0.024*P*)^2^ + 3.1419*P*] where *P* = (*F*_o_^2^ + 2*F*_c_^2^)/3
5544 reflections	(Δ/σ)_max_ = 0.005
262 parameters	Δρ_max_ = 0.44 e Å^−^^3^
0 restraints	Δρ_min_ = −0.58 e Å^−^^3^

### Special details

**Table:**

Experimental. The intensity data was collected on a Bruker Apex DUO 4 K CCD diffractometer using an exposure time of 40 s/frame. A total of 972 frames were collected with a frame width of 0.5° covering up to θ = 28.3° with 100% completeness accomplished.
Geometry. All e.s.d.'s (except the e.s.d. in the dihedral angle between two l.s. planes) are estimated using the full covariance matrix. The cell e.s.d.'s are taken into account individually in the estimation of e.s.d.'s in distances, angles and torsion angles; correlations between e.s.d.'s in cell parameters are only used when they are defined by crystal symmetry. An approximate (isotropic) treatment of cell e.s.d.'s is used for estimating e.s.d.'s involving l.s. planes.
Refinement. Refinement of *F*^2^ against ALL reflections. The weighted *R*-factor *wR* and goodness of fit *S* are based on *F*^2^, conventional *R*-factors *R* are based on *F*, with *F* set to zero for negative *F*^2^. The threshold expression of *F*^2^ > σ(*F*^2^) is used only for calculating *R*-factors(gt) *etc*. and is not relevant to the choice of reflections for refinement. *R*-factors based on *F*^2^ are statistically about twice as large as those based on *F*, and *R*- factors based on ALL data will be even larger.

### Fractional atomic coordinates and isotropic or equivalent isotropic displacement parameters (Å^2^)

**Table:**

	*x*	*y*	*z*	*U*_iso_*/*U*_eq_	
Ru1	0.192720 (13)	0.729519 (13)	1.097642 (10)	0.01308 (6)	
Cl1	0.31341 (4)	0.65099 (4)	1.13353 (3)	0.01973 (14)	
Cl2	0.12848 (4)	0.57711 (4)	1.09754 (3)	0.01605 (13)	
P1	0.22367 (4)	0.68784 (4)	0.97937 (3)	0.01223 (14)	
C1	0.1603 (2)	0.86916 (17)	1.06111 (14)	0.0241 (7)	
H1	0.1636	0.894	1.014	0.029*	
C2	0.22515 (19)	0.87954 (18)	1.10820 (14)	0.0240 (7)	
H2	0.2714	0.9128	1.0935	0.029*	
C3	0.22054 (19)	0.84011 (18)	1.17715 (14)	0.0224 (6)	
H3	0.2649	0.8444	1.2083	0.027*	
C4	0.15104 (19)	0.79438 (18)	1.20056 (14)	0.0232 (7)	
H4	0.1474	0.7705	1.2479	0.028*	
C5	0.08725 (19)	0.78461 (19)	1.15297 (15)	0.0260 (7)	
H5	0.0408	0.752	1.1679	0.031*	
C6	0.0910 (2)	0.82252 (19)	1.08314 (15)	0.0264 (7)	
H6	0.0471	0.8165	1.0515	0.032*	
C7	0.26500 (17)	0.56772 (16)	0.97117 (12)	0.0151 (6)	
H7A	0.3014	0.5571	1.012	0.018*	
H7B	0.2208	0.522	0.9759	0.018*	
C8	0.30938 (17)	0.54528 (16)	0.90309 (12)	0.0151 (5)	
C9	0.26901 (17)	0.51027 (16)	0.84315 (13)	0.0169 (6)	
H9	0.2131	0.5017	0.8449	0.02*	
C10	0.31070 (19)	0.48790 (17)	0.78088 (13)	0.0205 (6)	
H10	0.2833	0.4633	0.7405	0.025*	
C11	0.3918 (2)	0.50152 (18)	0.77798 (14)	0.0231 (7)	
H11	0.4199	0.4871	0.7353	0.028*	
C12	0.43254 (19)	0.53603 (19)	0.83694 (15)	0.0255 (7)	
H12	0.4884	0.545	0.8348	0.031*	
C13	0.39101 (18)	0.55751 (18)	0.89933 (14)	0.0210 (6)	
H13	0.4189	0.5808	0.9398	0.025*	
C14	0.29770 (16)	0.76094 (16)	0.93506 (12)	0.0131 (5)	
C15	0.29935 (17)	0.76972 (17)	0.86005 (12)	0.0169 (6)	
H15	0.2587	0.7413	0.8321	0.02*	
C16	0.35968 (17)	0.81950 (17)	0.82660 (13)	0.0166 (6)	
H16	0.3601	0.8257	0.776	0.02*	
C17	0.41946 (17)	0.86018 (17)	0.86719 (14)	0.0180 (6)	
H17	0.4605	0.8949	0.8443	0.022*	
C18	0.41973 (17)	0.85044 (17)	0.94114 (13)	0.0173 (6)	
H18	0.4615	0.8774	0.9686	0.021*	
C19	0.35912 (17)	0.80140 (17)	0.97485 (13)	0.0170 (6)	
H19	0.3594	0.7952	1.0255	0.02*	
C20	0.13550 (17)	0.69210 (17)	0.92313 (12)	0.0140 (5)	
C21	0.10851 (17)	0.77773 (17)	0.89283 (13)	0.0168 (6)	
H21	0.1402	0.8331	0.8966	0.02*	
C22	0.03634 (18)	0.78158 (19)	0.85764 (13)	0.0203 (6)	
H22	0.0186	0.8398	0.8381	0.024*	
C23	−0.01037 (18)	0.70135 (19)	0.85061 (13)	0.0198 (6)	
H23	−0.0597	0.7044	0.8261	0.024*	
C24	0.01564 (17)	0.61655 (18)	0.87968 (13)	0.0180 (6)	
H24	−0.0158	0.5612	0.8746	0.022*	
C25	0.08700 (17)	0.61219 (17)	0.91598 (12)	0.0154 (6)	
H25	0.1035	0.554	0.9365	0.018*	

### Atomic displacement parameters (Å^2^)

**Table:**

	*U*^11^	*U*^22^	*U*^33^	*U*^12^	*U*^13^	*U*^23^
Ru1	0.01380 (12)	0.01403 (9)	0.01141 (9)	−0.00318 (9)	0.00057 (9)	−0.00071 (7)
Cl1	0.0162 (4)	0.0272 (3)	0.0158 (3)	−0.0018 (3)	−0.0033 (3)	0.0008 (2)
Cl2	0.0149 (4)	0.0154 (3)	0.0178 (3)	−0.0033 (2)	0.0016 (3)	0.0020 (2)
P1	0.0122 (4)	0.0123 (3)	0.0122 (3)	−0.0016 (3)	−0.0003 (3)	0.0001 (2)
C1	0.040 (2)	0.0133 (12)	0.0190 (13)	0.0028 (12)	0.0008 (13)	−0.0013 (10)
C2	0.0279 (19)	0.0167 (12)	0.0275 (14)	−0.0081 (12)	0.0095 (13)	−0.0051 (10)
C3	0.0247 (19)	0.0203 (12)	0.0221 (13)	−0.0020 (12)	−0.0030 (12)	−0.0091 (10)
C4	0.031 (2)	0.0229 (13)	0.0159 (12)	−0.0034 (12)	0.0079 (12)	−0.0042 (10)
C5	0.0194 (18)	0.0239 (14)	0.0348 (15)	−0.0010 (13)	0.0085 (13)	−0.0097 (12)
C6	0.028 (2)	0.0224 (14)	0.0291 (15)	0.0073 (13)	−0.0067 (13)	−0.0119 (11)
C7	0.0163 (17)	0.0157 (11)	0.0131 (11)	0.0017 (10)	−0.0016 (11)	0.0004 (9)
C8	0.0186 (16)	0.0118 (10)	0.0151 (10)	0.0005 (11)	0.0002 (12)	0.0009 (9)
C9	0.0155 (17)	0.0139 (11)	0.0212 (12)	−0.0005 (11)	−0.0021 (11)	0.0003 (9)
C10	0.0289 (19)	0.0163 (12)	0.0164 (11)	0.0036 (12)	−0.0048 (13)	−0.0018 (9)
C11	0.031 (2)	0.0178 (12)	0.0207 (13)	0.0012 (12)	0.0110 (13)	−0.0011 (10)
C12	0.0176 (18)	0.0255 (14)	0.0335 (15)	−0.0042 (13)	0.0115 (14)	−0.0064 (12)
C13	0.0176 (17)	0.0218 (13)	0.0234 (13)	−0.0026 (11)	−0.0003 (13)	−0.0066 (11)
C14	0.0121 (16)	0.0135 (10)	0.0137 (10)	0.0012 (10)	−0.0003 (10)	0.0007 (9)
C15	0.0182 (17)	0.0170 (11)	0.0156 (11)	0.0012 (11)	−0.0015 (11)	−0.0012 (9)
C16	0.0161 (17)	0.0188 (12)	0.0151 (11)	0.0038 (11)	0.0032 (11)	0.0040 (10)
C17	0.0124 (16)	0.0166 (12)	0.0248 (13)	0.0017 (11)	0.0060 (12)	0.0040 (10)
C18	0.0132 (16)	0.0175 (12)	0.0211 (12)	−0.0015 (11)	−0.0037 (12)	−0.0013 (10)
C19	0.0158 (17)	0.0199 (12)	0.0154 (11)	0.0006 (11)	−0.0013 (11)	0.0004 (9)
C20	0.0141 (16)	0.0168 (11)	0.0111 (10)	−0.0025 (11)	0.0018 (10)	−0.0023 (9)
C21	0.0157 (16)	0.0151 (11)	0.0196 (12)	−0.0020 (11)	−0.0001 (11)	−0.0007 (10)
C22	0.0184 (17)	0.0208 (13)	0.0216 (12)	0.0044 (12)	−0.0030 (12)	0.0014 (10)
C23	0.0089 (16)	0.0291 (14)	0.0213 (13)	0.0005 (12)	−0.0033 (11)	−0.0032 (10)
C24	0.0143 (17)	0.0211 (12)	0.0186 (12)	−0.0035 (11)	0.0015 (12)	−0.0035 (10)
C25	0.0151 (16)	0.0156 (11)	0.0154 (11)	−0.0022 (11)	0.0011 (11)	−0.0007 (9)

### Geometric parameters (Å, º)

**Table:**

Ru1—C1	2.161 (3)	C9—H9	0.95
Ru1—C6	2.177 (3)	C10—C11	1.380 (4)
Ru1—C5	2.198 (3)	C10—H10	0.95
Ru1—C2	2.201 (3)	C11—C12	1.387 (4)
Ru1—C3	2.208 (2)	C11—H11	0.95
Ru1—C4	2.244 (3)	C12—C13	1.393 (4)
Ru1—P1	2.3466 (6)	C12—H12	0.95
Ru1—Cl1	2.4116 (7)	C13—H13	0.95
Ru1—Cl2	2.4127 (6)	C14—C19	1.397 (4)
P1—C14	1.819 (3)	C14—C15	1.408 (3)
P1—C20	1.820 (3)	C15—C16	1.385 (4)
P1—C7	1.843 (2)	C15—H15	0.95
C1—C6	1.403 (4)	C16—C17	1.386 (4)
C1—C2	1.410 (4)	C16—H16	0.95
C1—H1	0.95	C17—C18	1.389 (3)
C2—C3	1.406 (4)	C17—H17	0.95
C2—H2	0.95	C18—C19	1.386 (4)
C3—C4	1.407 (4)	C18—H18	0.95
C3—H3	0.95	C19—H19	0.95
C4—C5	1.402 (4)	C20—C25	1.401 (4)
C4—H4	0.95	C20—C21	1.413 (3)
C5—C6	1.413 (4)	C21—C22	1.383 (4)
C5—H5	0.95	C21—H21	0.95
C6—H6	0.95	C22—C23	1.387 (4)
C7—C8	1.510 (3)	C22—H22	0.95
C7—H7A	0.99	C23—C24	1.388 (4)
C7—H7B	0.99	C23—H23	0.95
C8—C13	1.387 (4)	C24—C25	1.382 (4)
C8—C9	1.401 (3)	C24—H24	0.95
C9—C10	1.396 (4)	C25—H25	0.95
			
C1—Ru1—C6	37.73 (11)	C4—C5—H5	119.5
C1—Ru1—C5	67.68 (11)	C6—C5—H5	119.5
C6—Ru1—C5	37.66 (10)	Ru1—C5—H5	129.1
C1—Ru1—C2	37.72 (11)	C1—C6—C5	119.1 (3)
C6—Ru1—C2	67.88 (12)	C1—C6—Ru1	70.48 (17)
C5—Ru1—C2	79.41 (11)	C5—C6—Ru1	71.96 (17)
C1—Ru1—C3	67.56 (10)	C1—C6—H6	120.4
C6—Ru1—C3	79.78 (11)	C5—C6—H6	120.4
C5—Ru1—C3	66.65 (11)	Ru1—C6—H6	129.4
C2—Ru1—C3	37.20 (9)	C8—C7—P1	116.81 (16)
C1—Ru1—C4	79.51 (10)	C8—C7—H7A	108.1
C6—Ru1—C4	67.26 (11)	P1—C7—H7A	108.1
C5—Ru1—C4	36.77 (11)	C8—C7—H7B	108.1
C2—Ru1—C4	66.81 (10)	P1—C7—H7B	108.1
C3—Ru1—C4	36.84 (10)	H7A—C7—H7B	107.3
C1—Ru1—P1	89.32 (7)	C13—C8—C9	119.0 (2)
C6—Ru1—P1	102.09 (7)	C13—C8—C7	120.5 (2)
C5—Ru1—P1	135.38 (8)	C9—C8—C7	120.5 (3)
C2—Ru1—P1	105.77 (7)	C10—C9—C8	120.2 (3)
C3—Ru1—P1	139.90 (8)	C10—C9—H9	119.9
C4—Ru1—P1	168.48 (8)	C8—C9—H9	119.9
C1—Ru1—Cl1	136.24 (9)	C11—C10—C9	119.9 (2)
C6—Ru1—Cl1	167.51 (7)	C11—C10—H10	120
C5—Ru1—Cl1	135.53 (8)	C9—C10—H10	120
C2—Ru1—Cl1	102.14 (9)	C10—C11—C12	120.5 (2)
C3—Ru1—Cl1	87.73 (8)	C10—C11—H11	119.7
C4—Ru1—Cl1	102.33 (8)	C12—C11—H11	119.7
P1—Ru1—Cl1	87.65 (2)	C11—C12—C13	119.5 (3)
C1—Ru1—Cl2	134.73 (9)	C11—C12—H12	120.2
C6—Ru1—Cl2	100.79 (8)	C13—C12—H12	120.2
C5—Ru1—Cl2	87.42 (8)	C8—C13—C12	120.8 (3)
C2—Ru1—Cl2	166.78 (8)	C8—C13—H13	119.6
C3—Ru1—Cl2	136.81 (7)	C12—C13—H13	119.6
C4—Ru1—Cl2	103.06 (7)	C19—C14—C15	118.6 (2)
P1—Ru1—Cl2	82.77 (2)	C19—C14—P1	119.88 (18)
Cl1—Ru1—Cl2	88.07 (2)	C15—C14—P1	121.1 (2)
C14—P1—C20	106.10 (11)	C16—C15—C14	120.6 (3)
C14—P1—C7	103.15 (12)	C16—C15—H15	119.7
C20—P1—C7	106.90 (12)	C14—C15—H15	119.7
C14—P1—Ru1	115.99 (8)	C15—C16—C17	119.8 (2)
C20—P1—Ru1	110.77 (8)	C15—C16—H16	120.1
C7—P1—Ru1	113.20 (8)	C17—C16—H16	120.1
C6—C1—C2	120.7 (3)	C16—C17—C18	120.4 (3)
C6—C1—Ru1	71.78 (16)	C16—C17—H17	119.8
C2—C1—Ru1	72.68 (15)	C18—C17—H17	119.8
C6—C1—H1	119.7	C19—C18—C17	120.0 (3)
C2—C1—H1	119.7	C19—C18—H18	120
Ru1—C1—H1	128.1	C17—C18—H18	120
C3—C2—C1	119.2 (3)	C18—C19—C14	120.6 (2)
C3—C2—Ru1	71.69 (15)	C18—C19—H19	119.7
C1—C2—Ru1	69.60 (15)	C14—C19—H19	119.7
C3—C2—H2	120.4	C25—C20—C21	117.8 (2)
C1—C2—H2	120.4	C25—C20—P1	120.25 (19)
Ru1—C2—H2	130.9	C21—C20—P1	121.50 (19)
C2—C3—C4	120.9 (3)	C22—C21—C20	120.5 (2)
C2—C3—Ru1	71.11 (15)	C22—C21—H21	119.8
C4—C3—Ru1	72.96 (15)	C20—C21—H21	119.8
C2—C3—H3	119.6	C21—C22—C23	120.7 (2)
C4—C3—H3	119.6	C21—C22—H22	119.6
Ru1—C3—H3	128.7	C23—C22—H22	119.6
C5—C4—C3	119.0 (3)	C22—C23—C24	119.4 (3)
C5—C4—Ru1	69.83 (15)	C22—C23—H23	120.3
C3—C4—Ru1	70.20 (14)	C24—C23—H23	120.3
C5—C4—H4	120.5	C25—C24—C23	120.4 (2)
C3—C4—H4	120.5	C25—C24—H24	119.8
Ru1—C4—H4	132.4	C23—C24—H24	119.8
C4—C5—C6	121.0 (3)	C24—C25—C20	121.2 (2)
C4—C5—Ru1	73.40 (18)	C24—C25—H25	119.4
C6—C5—Ru1	70.37 (17)	C20—C25—H25	119.4
			
C1—Ru1—P1—C14	57.32 (13)	C5—Ru1—C4—C3	−133.0 (3)
C6—Ru1—P1—C14	93.09 (12)	C2—Ru1—C4—C3	−29.14 (17)
C5—Ru1—P1—C14	113.77 (14)	P1—Ru1—C4—C3	−80.7 (5)
C2—Ru1—P1—C14	22.94 (13)	Cl1—Ru1—C4—C3	68.93 (17)
C3—Ru1—P1—C14	4.66 (16)	Cl2—Ru1—C4—C3	159.82 (16)
C4—Ru1—P1—C14	71.4 (4)	C3—C4—C5—C6	−2.1 (4)
Cl1—Ru1—P1—C14	−79.02 (9)	Ru1—C4—C5—C6	−54.0 (2)
Cl2—Ru1—P1—C14	−167.37 (10)	C3—C4—C5—Ru1	51.9 (2)
C1—Ru1—P1—C20	−63.67 (12)	C1—Ru1—C5—C4	−102.74 (18)
C6—Ru1—P1—C20	−27.90 (12)	C6—Ru1—C5—C4	−132.6 (2)
C5—Ru1—P1—C20	−7.22 (14)	C2—Ru1—C5—C4	−65.22 (17)
C2—Ru1—P1—C20	−98.05 (12)	C3—Ru1—C5—C4	−28.53 (16)
C3—Ru1—P1—C20	−116.32 (15)	P1—Ru1—C5—C4	−167.01 (12)
C4—Ru1—P1—C20	−49.6 (4)	Cl1—Ru1—C5—C4	31.4 (2)
Cl1—Ru1—P1—C20	160.00 (9)	Cl2—Ru1—C5—C4	115.99 (15)
Cl2—Ru1—P1—C20	71.65 (9)	C1—Ru1—C5—C6	29.85 (16)
C1—Ru1—P1—C7	176.28 (13)	C2—Ru1—C5—C6	67.37 (17)
C6—Ru1—P1—C7	−147.95 (13)	C3—Ru1—C5—C6	104.06 (18)
C5—Ru1—P1—C7	−127.27 (15)	C4—Ru1—C5—C6	132.6 (2)
C2—Ru1—P1—C7	141.91 (13)	P1—Ru1—C5—C6	−34.4 (2)
C3—Ru1—P1—C7	123.63 (16)	Cl1—Ru1—C5—C6	163.98 (13)
C4—Ru1—P1—C7	−169.6 (4)	Cl2—Ru1—C5—C6	−111.41 (16)
Cl1—Ru1—P1—C7	39.95 (10)	C2—C1—C6—C5	−1.0 (4)
Cl2—Ru1—P1—C7	−48.40 (10)	Ru1—C1—C6—C5	55.0 (2)
C5—Ru1—C1—C6	−29.80 (15)	C2—C1—C6—Ru1	−56.0 (2)
C2—Ru1—C1—C6	−131.7 (2)	C4—C5—C6—C1	1.1 (4)
C3—Ru1—C1—C6	−102.70 (17)	Ru1—C5—C6—C1	−54.3 (2)
C4—Ru1—C1—C6	−66.23 (16)	C4—C5—C6—Ru1	55.4 (2)
P1—Ru1—C1—C6	110.94 (15)	C5—Ru1—C6—C1	131.2 (2)
Cl1—Ru1—C1—C6	−163.16 (12)	C2—Ru1—C6—C1	29.54 (15)
Cl2—Ru1—C1—C6	31.82 (19)	C3—Ru1—C6—C1	66.38 (16)
C6—Ru1—C1—C2	131.7 (2)	C4—Ru1—C6—C1	102.65 (17)
C5—Ru1—C1—C2	101.90 (18)	P1—Ru1—C6—C1	−72.76 (15)
C3—Ru1—C1—C2	29.00 (16)	Cl1—Ru1—C6—C1	67.9 (4)
C4—Ru1—C1—C2	65.47 (17)	Cl2—Ru1—C6—C1	−157.58 (14)
P1—Ru1—C1—C2	−117.36 (16)	C1—Ru1—C6—C5	−131.2 (2)
Cl1—Ru1—C1—C2	−31.5 (2)	C2—Ru1—C6—C5	−101.65 (18)
Cl2—Ru1—C1—C2	163.52 (13)	C3—Ru1—C6—C5	−64.82 (17)
C6—C1—C2—C3	1.8 (4)	C4—Ru1—C6—C5	−28.54 (16)
Ru1—C1—C2—C3	−53.7 (2)	P1—Ru1—C6—C5	156.05 (15)
C6—C1—C2—Ru1	55.5 (2)	Cl1—Ru1—C6—C5	−63.3 (5)
C1—Ru1—C2—C3	132.2 (3)	Cl2—Ru1—C6—C5	71.22 (16)
C6—Ru1—C2—C3	102.62 (19)	C14—P1—C7—C8	−35.7 (2)
C5—Ru1—C2—C3	65.12 (19)	C20—P1—C7—C8	76.0 (2)
C4—Ru1—C2—C3	28.88 (18)	Ru1—P1—C7—C8	−161.80 (17)
P1—Ru1—C2—C3	−160.48 (16)	P1—C7—C8—C13	92.6 (3)
Cl1—Ru1—C2—C3	−69.50 (18)	P1—C7—C8—C9	−88.5 (3)
Cl2—Ru1—C2—C3	70.4 (4)	C13—C8—C9—C10	0.2 (3)
C6—Ru1—C2—C1	−29.55 (17)	C7—C8—C9—C10	−178.7 (2)
C5—Ru1—C2—C1	−67.05 (18)	C8—C9—C10—C11	−0.9 (4)
C3—Ru1—C2—C1	−132.2 (3)	C9—C10—C11—C12	1.0 (4)
C4—Ru1—C2—C1	−103.30 (19)	C10—C11—C12—C13	−0.4 (4)
P1—Ru1—C2—C1	67.34 (17)	C9—C8—C13—C12	0.4 (4)
Cl1—Ru1—C2—C1	158.33 (16)	C7—C8—C13—C12	179.3 (2)
Cl2—Ru1—C2—C1	−61.8 (4)	C11—C12—C13—C8	−0.3 (4)
C1—C2—C3—C4	−2.8 (4)	C20—P1—C14—C19	155.8 (2)
Ru1—C2—C3—C4	−55.6 (2)	C7—P1—C14—C19	−92.0 (2)
C1—C2—C3—Ru1	52.7 (2)	Ru1—P1—C14—C19	32.4 (2)
C1—Ru1—C3—C2	−29.38 (19)	C20—P1—C14—C15	−30.9 (2)
C6—Ru1—C3—C2	−66.73 (19)	C7—P1—C14—C15	81.3 (2)
C5—Ru1—C3—C2	−103.8 (2)	Ru1—P1—C14—C15	−154.40 (18)
C4—Ru1—C3—C2	−132.2 (3)	C19—C14—C15—C16	−1.6 (4)
P1—Ru1—C3—C2	29.9 (2)	P1—C14—C15—C16	−174.9 (2)
Cl1—Ru1—C3—C2	113.59 (18)	C14—C15—C16—C17	0.7 (4)
Cl2—Ru1—C3—C2	−161.65 (15)	C15—C16—C17—C18	0.7 (4)
C1—Ru1—C3—C4	102.9 (2)	C16—C17—C18—C19	−1.3 (4)
C6—Ru1—C3—C4	65.51 (18)	C17—C18—C19—C14	0.4 (4)
C5—Ru1—C3—C4	28.48 (17)	C15—C14—C19—C18	1.0 (4)
C2—Ru1—C3—C4	132.2 (3)	P1—C14—C19—C18	174.4 (2)
P1—Ru1—C3—C4	162.18 (13)	C14—P1—C20—C25	144.4 (2)
Cl1—Ru1—C3—C4	−114.17 (17)	C7—P1—C20—C25	34.8 (2)
Cl2—Ru1—C3—C4	−29.4 (2)	Ru1—P1—C20—C25	−88.9 (2)
C2—C3—C4—C5	3.0 (4)	C14—P1—C20—C21	−43.5 (2)
Ru1—C3—C4—C5	−51.8 (2)	C7—P1—C20—C21	−153.1 (2)
C2—C3—C4—Ru1	54.7 (2)	Ru1—P1—C20—C21	83.2 (2)
C1—Ru1—C4—C5	66.59 (18)	C25—C20—C21—C22	0.2 (4)
C6—Ru1—C4—C5	29.19 (17)	P1—C20—C21—C22	−172.1 (2)
C2—Ru1—C4—C5	103.85 (19)	C20—C21—C22—C23	−0.9 (4)
C3—Ru1—C4—C5	133.0 (3)	C21—C22—C23—C24	0.4 (4)
P1—Ru1—C4—C5	52.3 (5)	C22—C23—C24—C25	0.7 (4)
Cl1—Ru1—C4—C5	−158.07 (15)	C23—C24—C25—C20	−1.3 (4)
Cl2—Ru1—C4—C5	−67.19 (16)	C21—C20—C25—C24	0.9 (4)
C1—Ru1—C4—C3	−66.41 (18)	P1—C20—C25—C24	173.2 (2)
C6—Ru1—C4—C3	−103.80 (19)		

### Hydrogen-bond geometry (Å, º)

*Cg*1 and *Cg*2 are the centroids of the C8–C13 and C20–C25 rings, respectively.

**Table:**

*D*—H···*A*	*D*—H	H···*A*	*D*···*A*	*D*—H···*A*
C11—H11···Cl2^i^	0.95	2.85	3.568 (3)	133
C16—H16···Cl1^ii^	0.95	2.8	3.716 (3)	163
C2—H2···Cl2^iii^	0.95	2.87	3.733 (3)	151
C24—H24···Cl2^iv^	0.95	2.78	3.685 (3)	161
C21—H21···*Cg*1^v^	0.95	2.89	3.673 (3)	140
C4—H4···*Cg*2^vi^	0.95	3.00	3.789 (3)	141

Symmetry codes: (i) −*x*+1/2, −*y*+1, *z*−1/2; (ii) *x*, −*y*+3/2, *z*−1/2; (iii) −*x*+1/2, *y*+1/2, *z*; (iv) −*x*, −*y*+1, −*z*+2; (v) *x*, −*y*−3/2, *z*−1/2; (vi) *x*−1/2, *y*, −*z*−1/2.
